# Decreased long non‐coding RNA lincFOXF1 indicates poor progression and promotes cell migration and metastasis in osteosarcoma

**DOI:** 10.1111/jcmm.15828

**Published:** 2020-09-18

**Authors:** Shengquan Yang, Jian Chen, Bin Lv, Jun Zhang, Deli Li, Mengyuan Huang, Li Yuan, Guoyong Yin

**Affiliations:** ^1^ Department of Orthopaedics The First Affiliated Hospital of Nanjing Medical University Nanjing Jiangsu PR China; ^2^ Department of Orthopaedics The No. 1 People's Hospital of Yancheng Yancheng Jiangsu PR China; ^3^ Department of Biochemistry and Molecular Biology Nanjing Medical University Nanjing Jiangsu PR China

**Keywords:** EZH2, lincFOXF1, long non‐coding RNA, metastasis, migration, osteosarcoma

## Abstract

Long non‐coding RNAs have been demonstrated to be important regulators of various cancers, though the precise mechanisms remain unclear. Although lincFOXF1 has been reported to act as a tumour suppressor, its function and underlying mechanisms in osteosarcoma have not yet been explored. We employed quantitative real‐time polymerase chain reaction (qRT‐PCR) to evaluate the expression of lincFOXF1 and GAPDH in osteosarcoma tissues and cell lines, and colony‐formation, CCK8, wound‐healing, and transwell assays were conducted to analyse the proliferation, migration, and invasion capacity of osteosarcoma cells. Subcellular localization analysis by fractionation and RNA immunoprecipitation assays were performed to elucidate the mechanism responsible for lincFOXF1‐mediated phenotypes of osteosarcoma cells. The results revealed that lincFOXF1 expression is significantly decreased and strongly related to Enneking stage as well as metastasis in osteosarcoma patients. Further experiments showed that lincFOXF1 inhibits the migration, invasion and metastasis of cells in vitro and vivo. Mechanistic investigation demonstrated that lincFOXF1 physically binds to EZH2, a polycomb repressive complex 2 (PRC2) component, and a search for downstream targets suggested that G‐protein‐coupled receptor kinase‐interacting protein 1 (GIT1) is involved in the lincFOXF1‐mediated repression of osteosarcoma cells migration and invasion. Moreover, GIT1 expression is inversely correlated with lincFOXF1 in osteosarcoma. The present findings indicate that lincFOXF1 is involved in the progression of osteosarcoma through binding with EZH2, further regulating GIT1 expression. Our results suggest that lincFOXF1 may serve as a biomarker and therapeutic target for osteosarcoma patients.

## 1 INTRODUCTION

Osteosarcoma, also referred to as osteogenic sarcoma, is a prevalent malignant bone cancer that is primarily diagnosed in children and teenagers.^1^, ^2^ Osteosarcoma has a strong tendency to metastasize, and over 85% of metastatic disease involves the lung, contributing to the death of these patients.^3^ The introduction of neoadjuvant chemotherapy after the 1970s dramatically improved the long‐term survival rates of osteosarcoma patients from less than 20% to 65%‐70%.^4^ However, survival has remained virtually unchanged over the last three decades, and osteosarcoma patients with metastasis or relapse have a particularly poor survival of only 20%.^5^, ^6^ Osteosarcoma occurs sporadically, and its genetic and genomic landscapes are complex; thus, our current understanding of the molecular basis remains limited. Regardless, new therapies and molecular‐based research are urgently needed.

With the development of next‐generation sequencing and transcriptome analysis, an increasing number of studies are focusing on non‐coding RNAs, particularly long non‐coding RNAs (lncRNAs),^7^ which not only regulate cell development and differentiation^8^ but also contribute to various processes of tumour biology.^9^ Moreover, as lncRNAs are typically expressed in disease‐, tissue‐ and developmental stage‐specific manners, these molecules are appealing therapeutic targets and biomarkers in cancers.^10^, ^11^, ^12^

FOXF1‐adjacent non‐coding developmental regulatory RNA (lincFOXF1), an orthologous mouse lincFoxf1, was previously demonstrated to be essential for the normal development of the heart, body wall, and lungs as well as multiple other mesenchymal organs in mice.^13^, ^14^ The expression of lincFOXF1 is found in tissues derived from the lateral plate mesoderm^13^, ^14^; interestingly, the limb skeleton, in which osteosarcomas are primarily located, is also a product of the lateral plate mesoderm.^15^ Moreover, it has been reported that osteosarcoma may arise from mesenchymal stem cell‐derived osteoblasts.^16^, ^17^ Altogether, these findings prompted us to explore the role of lincFOXF1 in human osteosarcoma.

In the present study, the long non‐coding RNA lincFOXF1 was found to be significantly down‐regulated in osteosarcoma. Furthermore, the loss of lincFOXF1 expression was associated with clinicopathological features, and lincFOXF1 regulated the cell migration, invasion, and metastasis both in vitro and in vivo. Mechanistic analysis showed lincFOXF1 to be primarily located in the nucleus and to physically interact with EZH2. In addition, G‐protein‐coupled receptor kinase‐interacting protein 1 (GIT1) was strongly involved in the observed lincFOXF1‐mediated inhibition of cell migration and invasion. Collectively, these findings suggest that lincFOXF1 functions as a tumour suppressor in osteosarcoma and may be used as a predictive marker and therapeutic target.

## 2 MATERIALS AND METHODS

### 2.1 Microarray data analysis

Bioinformatics analyses were employed to assess expression of lincFOXF1 in sarcoma. Data for expression of sarcoma‐related genes as well as other genes were acquired from GSE17679 data sets. We also downloaded probe sequences from GEO or microarray manufacturers prior to using bowtie to reannotate probes consistent with the GENCODE Release 19 annotation for lncRNAs. We selected the probes with maximum signals to assess the expression of lncRNAs consistent with the corresponding gene.

### 2.2 Collection of tissues and ethics statement

We analysed 45 paired osteosarcoma tissues. The patients had undergone resection at the Department of Orthopedic Surgery, the First Affiliated Hospital of Nanjing Medical University and Yancheng First People's Hospital between September 2014 and October 2017. The Ethics Committee on Human Research of the Hospital approved the present study. We also obtained informed consent from the patients as well as their guardians prior to the collection of samples. Fresh tumour specimens were snap‐frozen in liquid nitrogen immediately after surgical resection, and formalin‐fixed, paraffin‐embedded surgical specimens were stored in the Department of Pathology.

### 2.3 Cell lines

We purchased the human osteosarcoma cell line 143B from ATCC (Manassas, USA) and obtained SAOS2, MG63, U2OS and MNNG/HOS Cl #5 cells and the normal human osteoblast cell line hFOB1.19 from Type Culture Collection of the Chinese Academy of Sciences (Shanghai, China). The cell lines were cultured in DMEM (Hyclone, South Logan, UT, USA) supplemented with 10% foetal bovine serum, 100 U/ml penicillin and streptomycin (Gibco, Carlsbad, CA, USA) at 37°C with 5% CO_2_.

### 2.4 RNA preparation and quantitative real‐time PCR

Total RNA was purified from osteosarcoma tissues and cells using Trizol reagent (Invitrogen, Carlsbad, USA). We synthesized cDNA and conducted real‐time RT‐PCR using SYBR® qPCR Kit (Takara, Japan) according to the manufacturer's instructions. The primers used in the present study are listed in Table S1.

### 2.5 Western blot analysis

Western blotting was performed with standard procedures. Protein concentrations were detected using a Bio‐Rad protein assay kit. A 50‐μg sample of protein extract was separated by 10% SDS‐PAGE and transferred to PVDF membranes (Millipore, USA). The membranes were then blocked in 5% non‐fat dry milk and incubated with the antibodies against GIT1 (1:1000, Abcam, ab153958) and GAPDH (1:5000, Bioworld, #AP0066). The membranes were incubated with a horseradish peroxidase‐conjugated secondary antibody for 1 hour at room temperature, and the protein bands were visualized using an electrochemiluminescence (ECL) chromogenic substrate and measured using densitometry (Quantity One software, Bio‐Rad).

### 2.6 Vector construction and cell transfection

We amplified the coding sequence of human lincFOXF1 and cloned it into the expression vector pcDNA3.1 (Invitrogen) according to the manufacturer's instructions. Small interfering RNA (siRNA) of lincFOXF1 was provided by Invitrogen and transfected into osteosarcoma cells using Lipofectamine 2000 (Invitrogen). All siRNA sequences are listed in Table S1.

### 2.7 Cell proliferation assay

We monitored cell viability using the Cell Counting Kit‐8 assay (CCK8) according to the manufacturer's instructions. Cell suspensions were seeded into 96‐well plates, cultured for the designated time (24, 48, 72 and 96 hours); the cells were incubated with CCK8 agent for another 4 hours, and absorbance was measured. For the colony‐formation assay, we plated cell suspensions in 6‐well plates in media supplemented with 10% FBS. The cells were fixed with methanol prior to staining with 0.1% crystal violet (Sigma‐Aldrich), and visible colonies were counted.

### 2.8 Wound‐healing assay

Cells were plated in 6‐well plates. Upon reaching 90% confluence, the surface of the cell layer was scratched using a sterile 200‐μl pipette tip, after which detached cells were removed by washing with culture medium. After culturing the cells in medium supplemented with 1% FBS, we obtained images in the same field at 0, 24, 36 and 48 hours using an inverted microscope (Olympus, Tokyo, Japan). The distance between the two edges of the scratch was measured using Digimizer image analysis software.

### 2.9 Boyden chamber migration and invasion assays

We performed cell migration and invasion assays with 24‐well transwell plates (8.0‐μm pore size, Millipore, Billerica, MA, USA) with or without Matrigel coating (Corning, NY, USA). Cells were cultured with serum‐free DMEM in the upper chamber, and DMEM supplemented with 10% FBS was added to the lower chamber. After 12 and 24 hours of incubation, we removed the residual cells in the upper chamber with cotton swabs and fixed the filters using 4% paraformaldehyde for 15 minutes prior to staining with 0.1% crystal violet. Cells in ten random optical fields from triplicate filters were counted and averaged.

### 2.10 Animal studies

We purchased male NOD/SCID mice (4‐6 weeks old) from the Animal Center of the Chinese Academy of Science (Shanghai, China) and maintained these animals in laminar flow cabinets within pathogen‐free settings. A total of 143B cells stably transfected with pcDNA‐lincFOXF1 or the empty vector were harvested and washed with PBS prior to suspension. After injecting the suspended cells into the lateral tail vein, the animals were monitored three times every week for an indication of morbidity associated with pulmonary metastasis. After 8 weeks, we sacrificed the mice by cervical dislocation and photographed the lungs to count the visible tumours on the lung surface. The lungs were fixed in 4% paraformaldehyde prior to embedding in paraffin. We stained the sections using haematoxylin and eosin (HE) to ascertain metastases by light microscopy. The procedure was approved by The Institutional Animal Care and Use Committee (IACUC) of Nanjing Medical University.

### 2.11 Subcellular fractionation location

We separated nuclear and cytosolic fractions using PARIS Kit (Life Technologies, USA) according to the manufacturer's instructions.

### 2.12 RNA immunoprecipitation assays

RNA immunoprecipitation (RIP) assays were performed with Magna RIP™ RNA‐Binding Protein Immunoprecipitation Kit (Millipore) according to the manufacturer's instructions. We obtained an anti‐EZH2 antibody for RIP tests from Millipore. After incubating RNase‐treated RIP lysates for 1 hour  at 37 °C, we detected co‐precipitated RNAs using RT‐PCR.

### 2.13 Statistical analysis

We conducted statistical assessments using SPSS 22.0 software (IBM, USA). We also estimated the significance of variations between groups using Student's *t* test or the *χ* ^2^ test, as required. Pearson's correlation was performed to examine correlation between lincFOXF1 and GIT1. Lastly, we calculated two‐sided *P*‐values, and a level of probability of 0.05 was considered statistically significant.

## 3 RESULTS

### 3.1 The antisense‐transcribed lncRNA lincFOXF1 was down‐regulated in osteosarcoma

To detect the lncRNA lincFOXF1 in soft‐tissue sarcomas, we performed an integrative analysis of TCGA sarcoma and GSE17679 microarray profiling using GEO data sets. The results confirmed lincFOXF1 to be strongly down‐regulated in sarcoma (*P* < 0.001; Figure A,B). To evaluate the expression levels of lincFOXF1 in osteosarcoma tissues, we first conducted quantitative polymerase chain reaction (qPCR) assays to examine 45 paired osteosarcoma and adjacent histologically normal tissues. The results showed that lincFOXF1 expression was substantially lower in tumour tissues (*P* < 0.05; Figure C). Subsequently, we assessed lincFOXF1 expression in the osteosarcoma cell lines SAOS2, MG63, MNNG‐HOS, U2OS and 143B cells and the normal human osteoblast cell line hFOB1.19. Remarkably decreased expression of lincFOXF1 was identified in osteosarcoma cells, especially in MNNG‐HOS and 143B cells, consistent with the results obtained for osteosarcoma tissues (Figure D). To further assess the relationship between lincFOXF1 expression and osteosarcoma clinicopathological features, osteosarcoma patients were separated into high‐ and low‐lincFOXF1 groups according to median values, and we found that lower lincFOXF1 expression was significantly correlated with Enneking stage (*P* = 0.042) and metastasis (*P* = 0.008) (Table 1). Nonetheless, we observed no significant relationship between lincFOXF1 expression and other factors, such as sex, age, location and tumour size. These data indicate that lincFOXF1 was significantly decreased in osteosarcoma and might contribute to the progression of this type of cancer.

**Figure 1:**
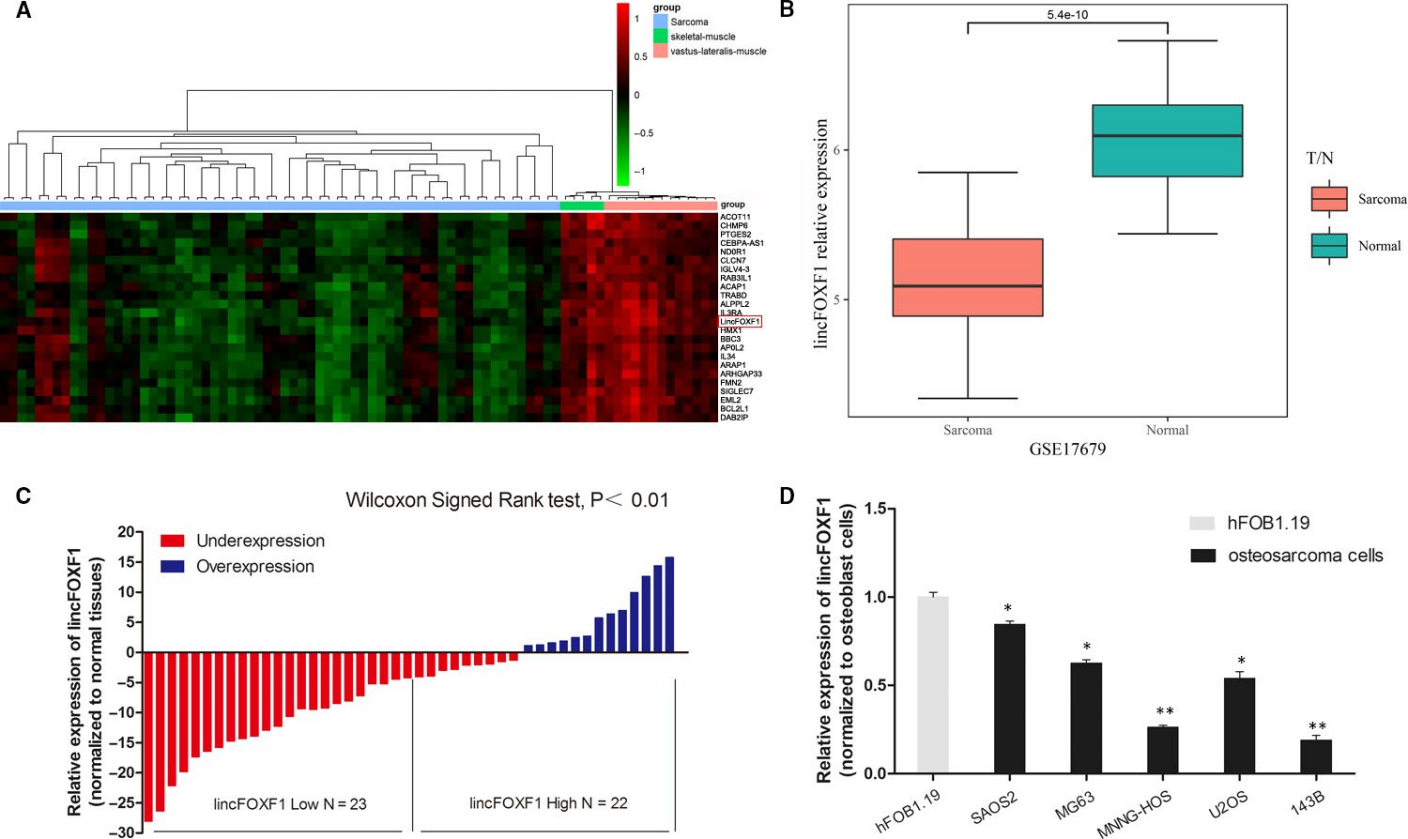
lncRNA lincFOXF1 is expressed at low levels in osteosarcoma tissues and cell lines. A, Hierarchical clustering analysis of differential expression of lincFOXF1 in sarcoma and normal tissues. B, lincFOXF1 is obviously decreased in sarcoma, as revealed by GSE17679 GEO data sets (*P* = 5.4e‐10). C, lincFOXF1 expression in osteosarcoma and adjacent non‐tumour tissues was analysed by qRT‐PCR (n = 45). D, lincFOXF1 expression in osteosarcoma cells, including SAOS2, MG63, MNNG/HOS Cl #5 (MNNG‐HOS), U2OS and 143B cells, compared with the normal osteoblast cell line hFOB1.19 was detected by qRT‐PCR. All experiments were performed in triplicate. Bars: SD; ^*^ *P* < 0.05 and ^**^ *P* < 0.01

**Table 1:** Association of long non‐coding RNA lincFOXF1 expression with the clinicopathological characteristics of 45 osteosarcoma patients

Clinical parameter	Expression of lincFOXF1		χ2 test *P*‐value*
	Low (n = 23)	High (n = 22)	
Sex			0.301
Male	14	10	
Female	9	12	
Age (y)			0.628
≤20	16	18	
21‐40	5	3	
>40	2	1	
Location			0.782
Distal femur	15	14	
Proximal tibia	4	6	
Proximal femur	2	1	
Proximal humerus	2	1	
Tumour size (involved bone)			0.102
≤1/3	7	12	
>1/3	16	10	
Enneking stage			0.042*
II A	1	6	
II B	15	14	
III	7	2	
Metastasis			0.008*
Yes	13	4	
No	10	18	

* *P* < 0.05.

### 3.2 lincFOXF1 exhibits no significant influence on osteosarcoma cell proliferation but represses osteosarcoma cell migration, invasion and metastasis

To analyse the functions of lincFOXF1 in osteosarcoma cells, we overexpressed or down‐regulated lincFOXF1 expression in MNNG‐HOS and 143B cells through transfection with the pcDNA3.1‐lincFOXF1 vector or lincFOXF1‐specific siRNA, respectively. According to qRT‐PCR, satisfactory transfection efficiency was achieved at 48 hours post transfection (Figure A). However, CCK8 and colony‐formation assay results indicated no significant impact of lincFOXF1 on MNNG‐HOS or 143B cell proliferation (Figure B,C). Subsequently, we performed a wound‐healing assay to evaluate the cell migration capacity. Interestingly, the results showed that knockdown of lincFOXF1 in MNNG‐HOS and 143B cells resulted in a markedly enhanced scratch‐closure rate (Figure D). Conversely, MNNG‐HOS and 143B cells exhibited reduced scratch closure after transfection with the pcDNA‐lincFOXF1. Consistently, transwell chamber cell migration assays showed that down‐regulation of lincFOXF1 facilitated migration, whereas overexpression of lincFOXF1 significantly inhibited migration (*P* < 0.01, Figure E). Next, we used transwell chambers coated with Matrigel to investigate the impact of lincFOXF1 on invasion. In contrast to the negative controls, down‐regulation of lincFOXF1 resulted in notably enhanced invasion (*P* < 0.05, Figure F), and overexpression of lincFOXF1 led to a reduction in invasion capacity (*P* < 0.05, Figure F). We also assessed the impact of lincFOXF1 on osteosarcoma metastasis in vivo: 143B cells stably transfected with pcDNA‐lincFOXF1 were injected into nude mice, and the number of metastatic nodules on the surface of lungs was determined after 8 weeks. We observed that compared with the control group, ectopic overexpression of lincFOXF1 led to obvious decreases in metastatic nodules (Figure G, *P* < 0.01). We confirmed this difference through analysis of whole lungs as well as by HE staining of lung sections (Figure G). Overall, the in vivo findings support the function of lincFOXF1 determined in vitro.

**Figure 2:**
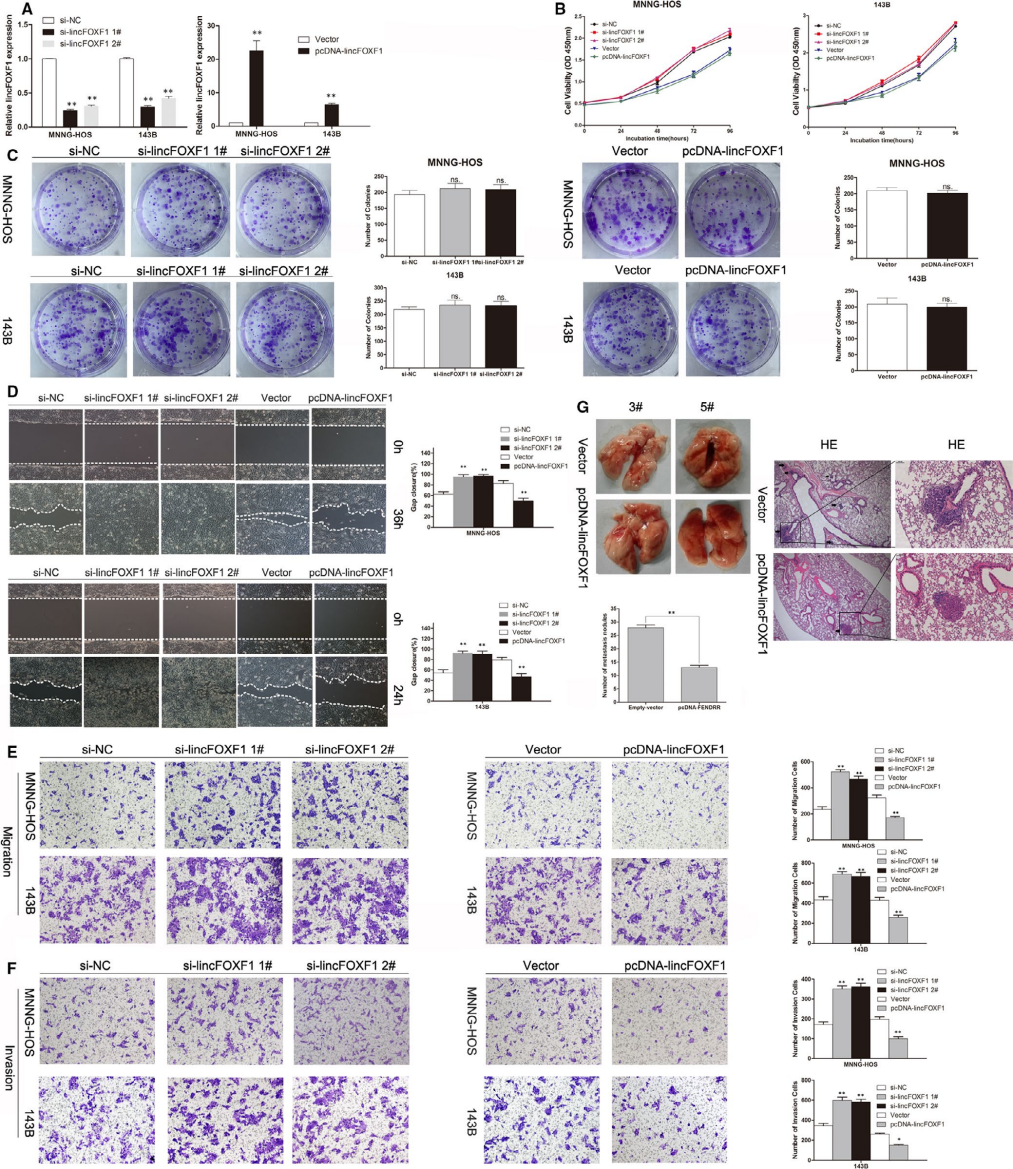
Effects of lincFOXF1 on osteosarcoma cell proliferation, migration and invasion in vitro and in vivo. A, lincFOXF1 expression in MNNG‐HOS and 143B cells transfected with pcDNA‐lincFOXF1 or lincFOXF1‐specific siRNA was detected by qRT‐PCR. B, CCK8 assays were used to determine the viability of transfected osteosarcoma cells. C, Colony‐formation assays were performed to assess the colony‐formation ability of si‐lincFOXF1‐ or pcDNA‐lincFOXF1‐transfected osteosarcoma cells. D, Wound‐healing assays were employed to investigate the migratory capacity of si‐lincFOXF1 or pcDNA‐lincFOXF1‐transfected osteosarcoma cells. (E) and (F) Transwell assays showed that lincFOXF1 knockdown promotes osteosarcoma cell migration and invasion and that lincFOXF1 overexpression inhibits osteosarcoma cell migration and invasion. G, lincFOXF1 inhibits osteosarcoma cell metastasis in vivo. All experiments were performed in triplicate. Bars: SD; ^*^ *P* < 0.05 and ^**^ *P* < 0.01

### 3.3 lincFOXF1 was primarily located in the nucleus and physically interacted with the PRC2 component EZH2

To further probe the mechanism responsible for lincFOXF1‐mediated phenotypes in osteosarcoma cells, we detected lincFOXF1 in nuclear and cytosolic fractions. The results showed that lincFOXF1 was primarily localized in the nucleus, and not in the cytoplasm (Figure A), suggesting that lincFOXF1 might play a regulatory role at the level of transcription. Indeed, a recent study confirmed a substantial number of lncRNA functions in collaboration with chromatin‐modification enzymes, suggesting epigenetic activation or silencing.^18^ In particular, PRC2 (Polycomb repressive complex 2), a methyltransferase comprising EZH2, SUZ12 and EDD‐1, catalyses the trimethylation of lysine residue 27 of histone 3 and suppresses the transcription of specific genes.^19^ A previous study also reported that lincFOXF1 interacts with PRC2 by binding to EZH2 to regulate target gene expression in human lung fibroblasts, human foot fibroblasts and HeLa cells.^18^ We performed an RNA immunoprecipitation assay to determine whether the function of lincFOXF1 in osteosarcoma is dependent on EZH2, and the results showed that lincFOXF1 bound tightly to EZH2 (Figure B). In addition, we performed rescue experiments to determine whether EZH2 is involved in the lincFOXF1‐induced decrease in osteosarcoma cell metastasis. Levels of expression were detected by qPCR after transfection with si‐EZH2 (Figure C); interestingly, we found that knock down of EZH2 slightly decreased osteosarcoma cells migration, which was consistent with the previous report.^20^ EZH2 was initially shown to have oncogenic properties; however, a large volume of data obtained in recent years have shown that PRC2 also has tumour‐suppressive functions.^21^ Studies suggested that it may be more beneficial to determine whether EZH2 is required for the development of the cancer cells, rather than modulating EZH2 levels to gauge its function as a tumour‐suppressor protein versus an oncoprotein.^22^ Migration assays revealed that the weakened phenotype induced by pcDNA‐lincFOXF1 was rescued after co‐transfection with si‐EZH2 (Figure D). Collectively, these findings indicate that lincFOXF1 physically interacts with EZH2 and EZH2 is required for lincFOXF1 medullated inhibition phenotypes which are involved in the metastasis of osteosarcoma cells.

**Figure 3:**
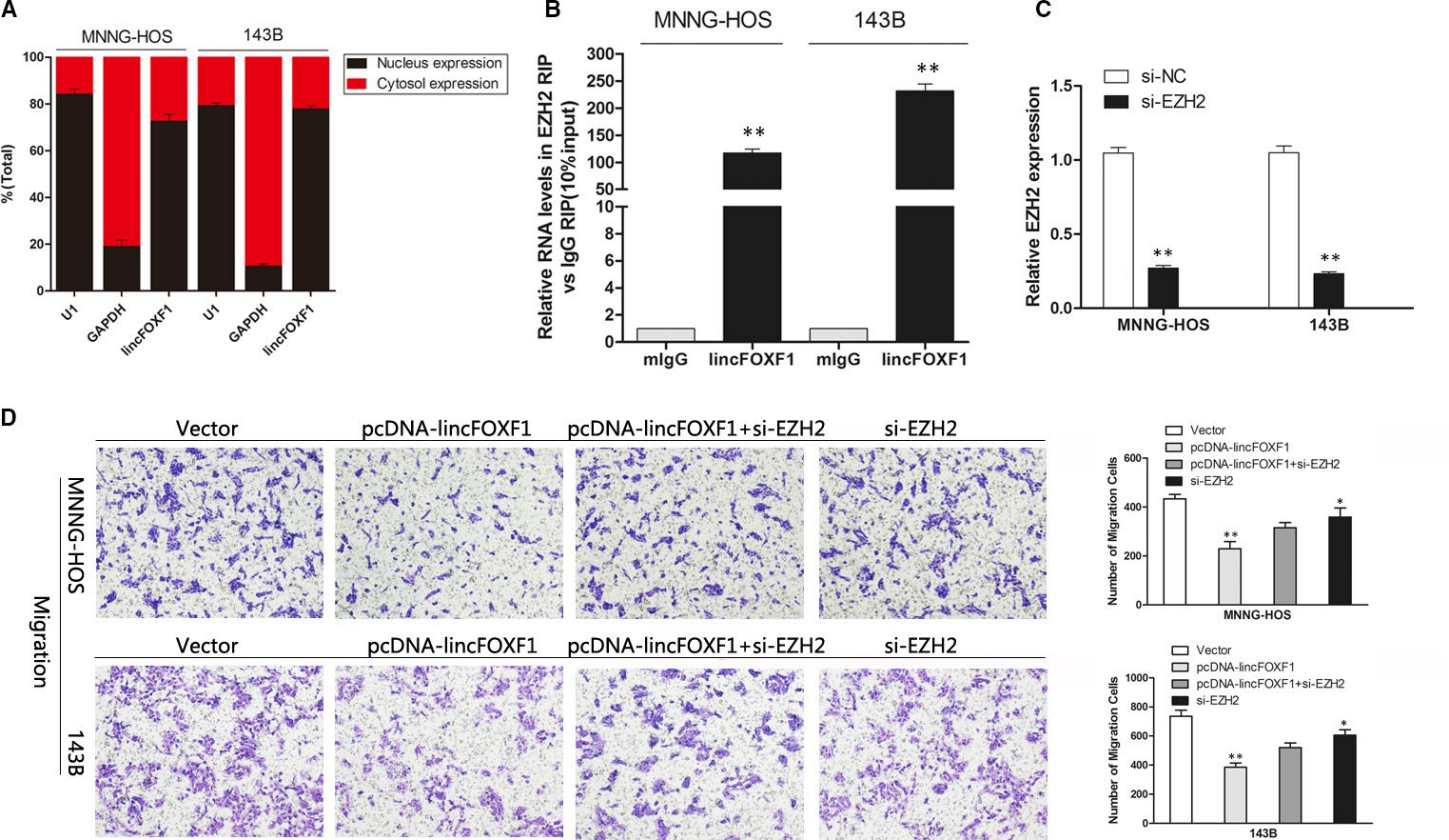
lincFOXF1 physically interacts with PRC2 components EZH2, and lincFOXF1‐induced inhibition of migration and invasion in osteosarcoma cells requires EZH2. A, RNA expression levels of nuclear and cytosolic fractions were measured by qRT‐PCR. GAPDH was used as a cytosolic marker, and U1 was used as a nuclear marker. B, RIP assays showed that lincFOXF1 binds to the EZH2 complex in MNNG‐HOS and 143B cells. C, EZH2 expression in MNNG‐HOS and 143B cells after transfection with si‐EZH2 was detected by qRT‐PCR. D, The effect of pcDNA‐lincFOXF1 expression and co‐treatment with si‐EZH2 on the migration of MNNG‐HOS and 143B cells was investigated by transwell assays, and overexpression of lincFOXF1 could not reduce the migration of osteosarcoma cells in the presence of EZH2 function blockade. All experiments were performed in triplicate. Bars: SD; ^*^ *P* < 0.05 and ^**^ *P* < 0.01

### 3.4 GIT1 may serve as a potential target involved in lincFOXF1‐mediated inhibition of osteosarcoma cell metastasis

Cell adhesion and extracellular matrix molecules have been implicated in the invasion and metastasis of various cancers. To identify the potential downstream target genes related to lincFOXF1 in osteosarcoma, we conducted qRT‐PCR to assess expression of multiple molecules (such as N‐cadherin, Integrin, CD44, ICAM‐1, Vimentin, Fibronectin, GIT1 and FOXF1) in osteosarcoma cells after gain or loss of lincFOXF1 function. Interestingly, GIT1 exhibited the most significant fold‐change among these molecules (Figure A,B): Overexpression or silencing of lincFOXF1 reduced GIT1 mRNA expression by 70% or elevated expression by 1.7‐fold respectively. As recent studies have revealed that GIT1 is related to tumour migration and invasion,^23^, ^24^ we analysed GIT1 protein expression using Western blot analysis and found an approximate 60% reduction and 1.4‐fold increase following transfection with pcDNA‐lincFOXF1 and si‐lincFOXF1 respectively (Figure C). Furthermore, we explored the association between GIT1 and lincFOXF1 expression in osteosarcoma tissues, and the results indicated an inverse correlation (Figure D, *R* = −0.451, *P* = 0.002). These data suggest that lincFOXF1 inhibition of osteosarcoma cell migration and invasion might rely partly on modulation of GIT1 expression.

**Figure 4:**
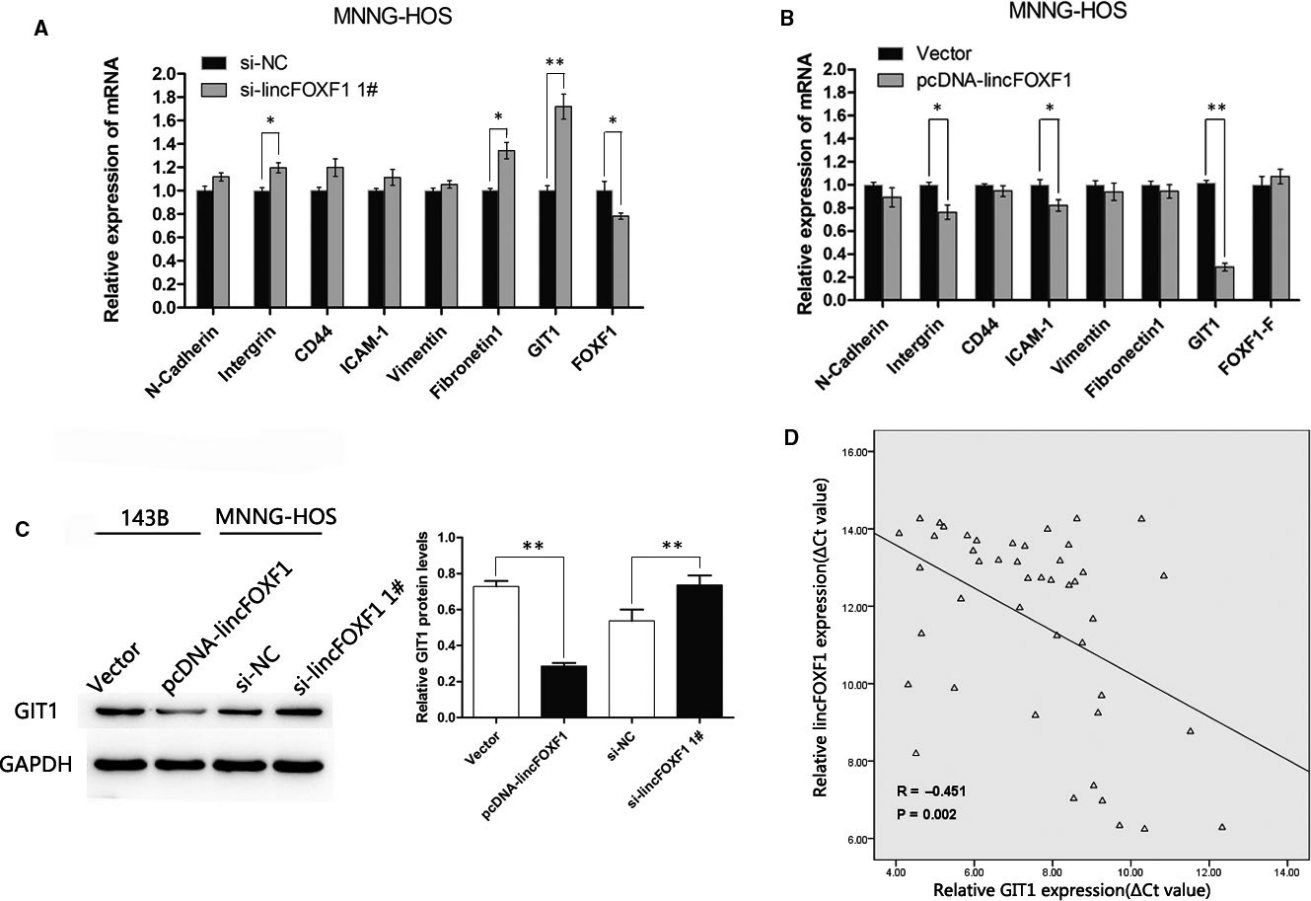
GIT1 is a potential target of lincFOXF1 and inversely correlated with lincFOXF1 expression in osteosarcoma tissues. (A) and (B) qRT‐PCR was used to detect expression of cell adhesion and extracellular matrix molecules, including N‐cadherin, Intergrin, CD44, ICAM‐1, Vimentin, Fibronectin, GIT1, and host gene FOXF1 after lincFOXF1 was knocked down or overexpressed in MNNG‐HOS cells. C, Western blot analyses were performed to confirm the gene expression in lincFOXF1‐overexpressing 143B cells or knocked down MNNG‐HOS cells compared with control cells. D, The inverse correlation between lincFOXF1 expression (ΔCt value) and GIT1 expression (ΔCt value) was analysed by qRT‐PCR in osteosarcoma tissues. All experiments were performed in triplicate. Bars: SD; ^*^ *P* < 0.05 and ^**^ *P* < 0.01

## 4 DISCUSSION

There is an increasing research focus on long non‐coding RNAs.^25^ Several well‐known lncRNAs, such as HOTAIR, H19, MALAT1 and TUG1, have been identified as functioning as oncogenes in osteosarcoma, whereas MEG3, GAS5 and TUSC7 have been proposed to act as tumour suppressors.^26^ Additionally, microarray and tiling array methods have been applied to study expression profiles of lncRNAs in osteosarcoma.^27^ However, the functional roles and precise mechanisms of lncRNAs in osteosarcoma remain unclear.^26^

In this study, we found lncRNA lincFOXF1 to be significantly down‐regulated in osteosarcoma, and this effect was associated with higher Enneking stage and lung metastasis. Silencing lincFOXF1 expression enhanced the cell migration and invasion, whereas ectopic expression of lincFOXF1 led to a significant inhibition of these capacities. Nonetheless, we found no apparent change in cellular proliferation after gain or loss of lincFOXF1 function, consistent with the findings of a previous study and clinical data showing that lincFOXF1 is not related to tumour size.^28^, ^29^ Conversely, a recent study^30^ reported that overexpression of lincFOXF1 repressed the cell cycle and promoted apoptosis in doxorubicin‐resilient osteosarcoma cells. The different results are likely due to the cell lines used as well as the context.

Furthermore, we isolated cytoplasmic and nuclear RNA of osteosarcoma cells and demonstrated that lincFOXF1 is primarily located in the nucleus. RIP assays showed that lincFOXF1 physically binds to EZH2. A major role for EZH2 in tumour cell proliferation and metastasis in cooperation with lncRNAs has been reported in various cancers,^31^, ^32^, ^33^, ^34^ though its association with lincFOXF1 in osteosarcoma cells is proposed herein for the first time. We also observed that the phenotype was rescued by si‐EZH2 co‐transfection. Moreover, we examined potential target genes related to the lincFOXF1 phenotype in osteosarcoma cells and interestingly found dramatic changes in GIT1 levels after overexpression or silencing of lincFOXF1.

GIT1 contains various domains, such as the ARFGAP, Spa2 homology, three ankyrin repeats, coiled‐coil and paxillin‐binding domains,^35^ which have a significant influence on cell migration,^36^ focal adhesion,^37^ lamellipodium formation, angiogenesis^38^ and endocytosis^39^; regulation of focal adhesion complexes involving FAK and paxillin are also involved in cancer cell invasion.^40^, ^41^, ^42^ Clinical evidence for GIT1’s involvement in human malignancies has been reported, suggesting that GIT1‐induced focal adhesion signalling is important for cancer progression and metastasis.^23^, ^24^, ^43^, ^44^, ^45^ In the present study, the level of GIT1 expression was up‐regulated in osteosarcoma patients and in opposite association with lincFOXF1. Small GTPases, such as Rac1/Cdc42, can regulate the formation of a multi‐subunit complex comprising GIT1, paxillin, PIX and PAK that promotes cell migration as well as lamellipodium formation.^46^ Thus, we propose that PIX and PAK levels and Rac1/cdc42 activities in GIT1‐induced metastatic osteosarcoma cells should be further investigated in subsequent studies.

In summary, the present results show that the long non‐coding RNA lincFOXF1 was significantly down‐regulated in osteosarcoma. Moreover, decreased lincFOXF1 expression was associated with tumour migration, invasion and metastasis. Additionally, mechanistic studies confirmed that lincFOXF1 binds to EZH2 and that blocking EZH2 expression partially reverses the lincFOXF1‐induced beneficial phenotype. GIT1 may constitute a target of lincFOXF1 in osteosarcoma. These results suggest that lincFOXF1 may serve as a biomarker and therapeutic target for osteosarcoma.

## CONFLICT OF INTEREST

The authors declare no conflicts of interest in this work.
