# Supplementary material for: Decreased long non‐coding RNA lincFOXF1 indicates poor progression and promotes cell migration and metastasis in osteosarcoma
Source: J Cell Mol Med. 2020 Sep 18;24(21):12633–41. doi: 10.1111/jcmm.15828 (PMC7686999; doi:10.1111/jcmm.15828)
Supplement: Supplementary file 1 [file JCMM-24-12633-s001.docx]

| **Suppl. Table S1. The list of primers and siRNA sequence.** | | | | | | | | | | | |  |
| --- | --- | --- | --- | --- | --- | --- | --- | --- | --- | --- | --- | --- |
| GENE | Forward primer | Reverse Primer |  |  |  | |  | |  |  |  |  |
| Human primers used for qRT-PCR | | | | | |  | |  | |  |  | |
| lincFOXF1 | TAAAATTGCAGATCCTCCG | AACGTTCGCATTGGTTTAGC | | | |  | |  | |  |  | |
| N-cadherin | TCTGGGTCTGTTTTATTACTCCTGG | CGAGCTGATGACAAATAGCGG | | | |  | |  | |  |  | |
| Integrinbeta 1 | CCTACTTCTGCACGATGTGATG | CCTTTGCTACGGTTGGTTACATT | | | |  | |  | |  |  | |
| CD44 | CTGCCGCTTTGCAGGTGTA | CATTGTGGGCAAGGTGCTATT | | | |  | |  | |  |  | |
| ICAM-1 | GTGACCAGCCCAAGTTGTTG | AGTCCAGTACACGGTGAGGA | | | |  | |  | |  |  | |
| Vimentin | AACTTAGGGGCGCTCTTGTC | CCTGCTGTCCCGCCG | | | |  | |  | |  |  | |
| Fibronectin1 | GGTGACACTTATGAGCGTCCTAAA | AACATGTAACCACCAGTCTCATGTG | | | |  | |  | |  |  | |
| GIT1 | GCCGACAGTGACTATGAGAA | AGTTCCTGAATGTTCTTGGT | | | |  | |  | |  |  | |
| FOXF1 | CACTCCCTGGAGCAGCCGTATC | AAGGCTTGATGTCTTGGTAGGTGA | | | |  | |  | |  |  | |
| GAPDH | GCTCTCTGCTCCTCCTGTTC | ACGACCAAATCCGTTGACTC | | | |  | |  | |  |  | |
| U1 | GGGAGATACCATGATCACGAAGGT | CCACAAATTATGCAGTCGAGTTTCCC | | | |  | |  | |  |  | |
| Sequences for siRNAs | | | | | |  | |  | |  |  | |
| si-lincFOXF1 1# | CCAGCCAUGUGAUUCCAAATT | UUUGGAAUCACAUGGCUGGTT | | | |  | |  | |  |  | |
| si-lincFOXF1 2# | GCGAUUGACUGUCUUAUAATT | UUAUAAGACAGUCAAUCGCTT | | | |  | |  | |  |  | |
| si-EZH2 | GAGGUUCAGACGAGCUGAUUU | AUCAGCUCGUCUGAACCUCUU | | | |  | |  | |  |  | |
